# Urine 3-Nitrotyrosine and Serum HDL as Potential Biomarkers of Depression

**DOI:** 10.3390/jcm12010377

**Published:** 2023-01-03

**Authors:** Aleksander Nobis, Daniel Zalewski, Eliza Samaryn, Mateusz Maciejczyk, Anna Zalewska, Napoleon Waszkiewicz

**Affiliations:** 1 Department of Psychiatry, Medical University of Białystok, ul. Wołodyjowskiego 2, 15-369 Białystok, Poland; 2 Department of Hygiene, Epidemiology, and Ergonomics, Medical University of Białystok, 15-089 Białystok, Poland; 3 Department of Restorative Dentistry, Medical University of Białystok, ul. M. Skłodowskiej-Curie 24A, 15-276 Białystok, Poland

**Keywords:** 3-nitrotyrosine, oxidative stress, HDL, biomarker, marker, depression, MDD, urine, cholesterol, β-amyloid

## Abstract

Depression (MDD) is a leading psychiatric entity worldwide, with a high impact on individual life and public health. In recent years, efforts have been made to elucidate its biological underpinnings. MDD biomarker research provides promise for a better understanding of the biochemical processes involved in its pathogenesis. Oxidative and nitrosative stress (O&NS) and lipid disturbances are reported as major factors favoring the occurrence of depression. A total of 29 patients with MDD and 30 healthy volunteers were examined using the Hamilton Depression Scale (HAM-D), the Hamilton Anxiety Scale (HAM-A), and the Beck Depression Inventory (BDI). Blood and urine were collected to search for potential MDD biomarkers. O&NS parameters and β-amyloid were assessed in the urine, while cholesterol fractions were assessed in the blood. The group of depressed patients was characterized by higher concentrations of urine superoxide dismutase (SOD), 3-nitrotyrosine (3-NT), catalase (CAT), reduced glutathione (GSH), tryptophan (TRY), and serum triglycerides (TGA), along with lower levels of serum high-density lipoprotein (HDL). Elevated urine 3-NT and decreased serum HDL, considered together, were found to have the greatest potential as markers of depression. The study supports the importance of oxidative stress and cholesterol disturbances in MDD. Further research is required to assess their clinical usefulness as markers.

## 1. Introduction

Depression (MDD) is a leading psychiatric entity, affecting 350 million people worldwide, i.e., 5% of the global population [1]. It is characterized by a high suicide rate and a high number of disability-adjusted life years, and it has a substantial impact on a patient’s quality of life [2]. MDD is clinically characterized by lowered mood, lack of energy, anhedonia, sleep and appetite disturbances, problems with concentration, decreased libido, and suicidal thoughts. Nevertheless, the clinical image of MDD may vary considerably from person to person, so much so that several subtypes of depression have been described. MDD is a complex and heterogeneous disorder with multiple psychological, social, and biological factors involved in its etiopathogenesis. Genetic predisposition, monoamine deficiency, stress axis dysregulation, and pro-inflammatory state are among the major biological factors enhancing depression development. Oxidative and nitrosative stress (O&NS), closely interrelated with the inflammatory processes, are also reported as pivotal biological factors involved in depression [3]. Oxidative stress is a persistent imbalance between the antioxidant defense system and oxidative damage, caused by reactive oxygen species (ROS), which are a side product of the mitochondrial oxidative chain. The main ROS include hydrogen peroxide, hydroxy radical, and superoxide anion [4]. Interestingly, the brain is particularly vulnerable to oxidative damage due to high oxygen consumption per weight unit, high concentration of unsaturated fatty acids, and oxidative defense system paucity. Nitrosative stress is a measure of the detrimental effect exerted on body cells by reactive nitrogen species (RNS). Examples of RNS are nitric oxide and peroxynitrite. ROS/RNS were found in the brain tissue of depressed patients in a post-mortem study [5]. Under physiological conditions, ROS/RNS are neutralized by several anti-oxidant defense pathways, including ROS/RNS scavenging (e.g., glutathione) or enzymatic deactivation (e.g., superoxide dismutase, glutathione peroxidase, and catalase). There are also ways through which oxidative stress favors anti-oxidant processes activation (e.g., an increase in Nrf-2 levels, which itself promotes antioxidant genes transcription) [6]. The interplay between pro- and antioxidant systems in depression has been described in detail by Maes and Carvalho [7]. When not efficiently counterbalanced by anti-oxidant systems, ROS/RNS damage cellular macromolecules, such as proteins, lipids, and DNA, leading to a formation of so-called neo-epitopes, which increase the autoimmune response. Such damages have been proved in MDD [8]. There is evidence that MDD is characterized by a neurodegenerative process [9], and O&NS pathways, when activated, favor neurodegeneration through different mechanisms, including direct ROS/RNS detrimental effect, enhanced neuroinflammation, and neurotoxic effects of by-products of the ROS-activated kynurenine pathway—quinolinic acid (QA) and 3-hydroxykynurenine (3-HKN) [10]. Interestingly, the activation of indoleamine 2,3-dioxygenase (IDO)—an enzyme of the kynurenine pathway—is likely the means through which inflammation induces depression [11]. Oxidative stress and neurodegeneration are closely interrelated. Oxidative stress is involved in the etiology of neurodegenerative disorders such as Alzheimer’s disease (AD). In AD, redox active metal ions (e.g., copper, iron, or zinc) aggregate with β-amyloid peptide—the hallmark of AD. When bound, they can promote ROS production and enhance the oxidative damage of the β-amyloid peptide itself, as well as other surrounding molecules, such as lipids or proteins [12,13]. Interestingly, MDD is known to be a prodrome and a risk factor of AD. Moreover, there is a certain overlap in behavioral signs of MDD and AD [14]. That is why β-amyloid was investigated as potentially altered in MDD, especially in older patients, with conflicting results [15,16]. In the majority of PET studies, higher levels of cortical β-amyloid deposition were found in elderly depressive patients [17,18,19], while contradictory results have also been reported [20,21].

The psychiatric diagnostic process, unlike the process in many other medical fields, remains mainly symptom-based. Today’s psychiatry research, tending toward more objective disease diagnosis and more personalized treatment, focuses on disease biomarkers. The better understanding of underlying pathophysiological processes may lead to a more accurate diagnosis and better-tailored treatment. An increasing body of evidence suggests the existence of several biochemical markers of MDD, able to differentiate between depressive patients and the healthy population [22,23]. Some of them have the potential to monitor treatment response or to predict the increased risk of depression incidence [24]. Oxidative stress can be evidenced by the presence of oxidative stress biomarkers, which may be found in any body fluid. The majority of MDD biomarker studies focus on blood biomarkers. For example, the umbrella meta-analysis of Carvalho et al. [25] points at some promising biomarkers of depression: interleukin-6, C-reactive protein, tumor necrosis factor-α, malondialdehyde, F2-isoprostanes, glutamate, total cholesterol, brain-derived neurotrophic factor, fibroblast growth factor-2, and insulin-like growth factor-1. Urine biomarkers are an interesting alternative to those of the blood, as urine collection is easy and non-invasive, and urine biomarkers concentrations may at least partially reflect their concentration in the blood. Urinary MDD biomarkers have also been assessed in some studies. For example, Chen et al. [23] discovered a metabolomic biomarker panel for diagnosing MDD consisting of four metabolites: aminomalonic acid, N-methylnicotinamide, hippuric acid, and azelaic acid. Van Buel et al. [26] found a set of 9 serum and 8 urine biomarkers correlating with depression.

This study aimed to assess urine oxidative stress parameters and β-amyloid concentration in the group of patients with MDD diagnosis in comparison to the healthy volunteers group to find out if they are associated with MDD and if any of them could potentially serve as MDD biomarkers.

## 2. Materials and Methods

### 2.1. Study Population

The study was conducted in the Department of Psychiatry at the Medical University of Białystok. Study participants were recruited from inpatients hospitalized with the diagnosis of unipolar depression between December 2021 and July 2022 in the Dr. Stanisław Deresz Independent Public Psychiatric Health Care Centre in Choroszcz, Poland. A total of 46 patients were eligible to participate in the study, and 17 of them eventually did not meet the inclusion criteria for one of the following reasons: lack of consent, or considerable psychiatric or somatic comorbidity. A total of 29 patients (11 men, 18 women), aged 18–65, were recruited into the study. The mean age was 43.3 years. All patients were Polish, and of Caucasian race. In the study group, there were 4 (13.8%) patients presenting with their first MDD episode and 25 (86.2%) presenting with a subsequent episode. Pregnant and breastfeeding women, and patients with obesity, diabetes, and inflammatory, autoimmune, and endocrine diseases, were excluded. None of the patients were drug-naïve at the recruitment. They received drugs from SSRI or SNRI groups or vortioxetine. The control group was recruited from among healthy volunteers, selected by sex and age to match those in the study group. The control group consisted of 30 people (10 men, 20 women), with a mean age of 41.8 years. The study design was accepted by the Ethics Committee of the Medical University of Białystok (permission: RI-002/582/2019) and was carried out in accordance with the Guidelines for Good Clinical Practice and the Helsinki Declaration. All study participants signed informed consent forms.

### 2.2. Study Design

The diagnosis of depression (i.e., depressive episode or recurrent depressive disorder) was based on ICD-11 criteria and confirmed by an experienced psychiatrist (N.W.). The MINI psychiatric interview was used in order to exclude other potential psychiatric entities [27]. Patients qualified in this way were then assessed with the use of the Beck Depression Inventory (BDI) [28], the Hamilton Depression Scale (HAM-D) [29], and the Hamilton Anxiety Scale (HAM-A) [30]. The persistence of depression was measured as the number of years since the beginning of the first depressive episode.

A blood analysis (complete blood count, potassium, sodium, creatinine, AlAT, AspAT, CRP, TSH, total cholesterol, LDL, HDL) was performed for each study participant before inclusion into the study in order to assess the general health of each individual. Blood analyses were performed on the Cobas Integra 400+ analyzer (Roche), using the immunoturbidimetric method for CRP determinations, and the enzymatic-colorimetric method for total cholesterol and HDL determinations. The LDL concentration was calculated using the Friedewald formula.

Urine samples were collected from the midstream of the first-morning urine and centrifuged at 1300× *g* for 10 min at 4 °C. (MPW 351, MPW Med. Instruments, Warsaw, Poland). The supernatant was collected, frozen, and stored at −80 °C in Eppendorf tubes until the biochemical analysis was performed.

### 2.3. Biochemical Procedures

All samples were then simultaneously tested. The following parameters were assessed: Total antioxidant capacity (TAC), catalase (CAT), glutathione peroxidase (GPx), superoxide dismutase (SOD), reduced glutathione (GSH), total oxidant status (TOS), 3-nitrotyrosine (3-NT), advanced glycoxidation end products (AGEs), advanced oxidation protein products (AOPP), N-formylkynurenine (NFKN), kynurenine (KN), tryptophan (TRY), and β-amyloid.

All reagents for the biochemical assays were obtained from Sigma-Aldrich (Saint Louis, MO, USA). The list of reagent catalog numbers is listed in the Supplementary Materials. The fluorescence/absorbance was measured using an Infinite M200 PRO Multimode Microplate Reader, Tecan (Tecan Group Ltd., Männedorf, Switzerland). All determinations were standardized to 1 mg of total protein.

#### 2.3.1. Anti-Oxidant Defense Systems Assays

Catalase (CAT) activity was assessed spectrophotometrically by measuring the decomposition rate of hydrogen peroxide. The absorbance was measured at 240 nm [31]. It was assumed that 1 unit of CAT decomposes 1 mmol hydrogen peroxide per 1 minute.

Superoxide dismutase-1 (SOD) activity was assayed spectrophotometrically by measuring the inhibition of adrenaline oxidation to adrenochrome at 480 nm [32]. A quantity of 1 unit of SOD activity was defined as a quantity of enzyme necessary to inhibit the oxidation of adrenaline by 50%.

Glutathione peroxidase (GPx) activity was assessed spectrophotometrically in the presence of NADPH and glutathione reductase, based on the reduction of organic peroxides. The absorbance was estimated at 340 nm [33]. A quantity of 1 unit of GPx activity was defined as catalyzing the oxidation of 1 μmol of NADPH per 1 minute.

Reduced glutathione (GSH) concentration was assessed spectrophotometrically based on the reaction with 5,5′-dithiobis-2-nitrobenzoic acid (DTNB). The absorbance of the resulting complex was estimated at 412 nm [34]. The standard curve for GSH (0–50 umol/L) was used.

Total antioxidant capacity (TAC) levels were estimated spectrophotometrically based on the reaction with 2,2-azinobis-3-ethylbenzothiazoline-6-sulfonic acid radical cation (ABTS*+) [35]. Absorbance changes were measured at 660 nm. TAC levels were calculated with the use of the calibration curve for 6-hydroxy-2,5,7,8-tetramethylchroman-2-carboxylic acid (Trolox; 0–3 mmol/L).

#### 2.3.2. Oxidative and Nitrosative Damage Assays

The content of advanced oxidation protein product (AOPP) was estimated spectrophotometrically at 340 nm with the use of the iodine ion by measuring its oxidative capacity. For AOPP assessment, urine samples were diluted 1:50 (*v*/*v*) in phosphate-buffered saline, pH 7.2 [36]. The concentration of AOPP was calculated from the standard curve for chloramine T (0–100 umol/L).

The presence of advanced glycation end-products (AGE) was assessed fluorometrically by measuring AGE-specific fluorescence at 350 nm/440 nm. For AGE assessment, urine samples were diluted 1:50 (*v*/*v*) in phosphate-buffered saline, pH 7.2 [36].

For KN, NFKN, TRY, and 3-NT determinations, urine samples were diluted (1:10, *v*/*v*) in 0.1 M H_2_SO_4_. Fluorescence was analyzed at 365/480, 325/434, 95/340, and 330/415 nm, respectively, [37], and all results were normalized to a fluorescence of 0.1 mg/mL quinine sulfate (in 0.1 M H_2_SO_4_) [38].

The level of TOS was determined colorimetrically based on the oxidation of Fe^2+^ to Fe^3+^ ions in the presence of the oxidants in the sample [35]. The absorbance was measured bichromatically at 560/800 nm. The TOS level was calculated from the standard curve for H_2_O_2_ (0–200 umol/L).

The content of total protein in the samples was estimated by the bicinchoninic acid (BCA) method [39] using the commercial kit-Thermo Scientific Pierce BCA Protein Assay (Rockford, IL, USA), in accordance with the manufacturer’s instructions.

The formation of beta-amyloid was evaluated by adding 10 uL of Thioflavin T to 90 uL of the urine sample. Thioflavin T fluorescence was measured at 385/485 nm [40].

### 2.4. Statistical Analysis

The significance level of statistical tests in the analysis was set at α = 0.05. Testing of variables on a continuous scale was performed using the Shapiro–Wilk test.

Two types of tests were used: parametric (for normally distributed variables) and nonparametric (for nonnormally distributed variables).

For normally distributed variables, the distribution measures of central tendency were given in terms of *M* (*SD*) and *Mdn* (*Q1–Q3*) for non-normally distributed variables.

The Mann–Whitney test was used to compare the means of two independent groups with nonnormal distributions. The effect size measure was estimated using the ${\hat{r}}_{biserial}^{rank}$. Interpretation of the effect size was based on Funder’s convention [41].

The Welch’s *t*-test was used to compare the means of two independent groups with normal distribution. The effect size was estimated using the ${\hat{g}}_{Hedges}$. Interpretation of the ${\hat{g}}_{Hedges}\;$ effect size was based on the Cohen convention [42].

The relationships between the two nominal variables were estimated using Pearson’s Chi-square test and Fisher’s exact test. A measure of the strength of the relationship, phi (φ), was calculated in the case of *df* = 1 and Cramer’s *V,* in the case of *df* > 1.

In the case of test significance under the condition *df* > 1, equality between pairs of groups was estimated by a post hoc test using the “*BH*” method (also known as “fdr”), which controls for false discovery rate, i.e., the expected proportion of false discoveries among the rejected hypotheses [43]. The effect size of the test was also interpreted according to the Funder’s convention.

The correlation of two independent variables on a continuous or quotient scale, corresponding to the assumptions of normality with *n* > 30, was calculated using Pearson’s method [44], *r_p_*. The test statistic followed a t-distribution, with (x-2) degrees of freedom.

For non-normal distributions or in the case of a small sample size (*n* ≤ 30), the Spearman method was used. The *p* values were calculated using the asymptotic *t* approximation.

The multivariate effect analysis of the selected clinical parameters on the factor depression was studied with the logistic regression model, based on a generalized linear model. The iterative weighted least squares (IWLS) method was used to fit the model. The description of the error distribution and the joint function was designed based on Gaussian family objects.

The logit of the unknown probability of depression *p_i_* was modeled as a linear function of predictors *X*_i_ based on formula (1):

(1) $$logit\left(p_{i}\right)=ln(\frac{p_{i}}{1-p_{i}})=\beta 0+\beta_{1}\cdot x_{1,i}+\ldots +\beta_{k}\cdot x_{k,i}$$

where the observed values *Yi*~*binomial*, with *p* = *p_i_* for a given *x_i_* and *n* = *k* for binary responses.

Coefficients β_j_ (*j* = 1, …*k*) were estimated using the maximum likelihood method.

The interpretation of the parameter β_j_ was the additive effect that a unit change in the *j* variable has on the odds ratio, defined according to Equation (2):

(2) $$OR_{A\times B}=\frac{S\left(A\right)}{S\left(B\right)}=\frac{\frac{P\left(A\right)}{1-P\left(A\right)}}{\frac{P\left(B\right)}{1-P\left(B\right)}}=\frac{P\left(A\right)\times \left(1-P\left(B\right)\right)}{P\left(B\right)\times \left(1-P\left(A\right)\right)}{\;}_{\;}$$

where *A, B*—study groups, *P*—probability of occurrence of depression in a group, and *S*—chance of occurrence of depression in a group [45].

The form of the implemented regression model is expressed by Equation (3):

(3) $$p_{i}=\frac{1}{1+e^{-\left(\beta 0+\beta 1\times 1,i+\ldots +\beta k\times k,i\right)}}$$

Standardized parameters were obtained by fitting the model on a standardized version of the dataset. The 95% confidence intervals (*CIs*) and the *p*-values were computed using a Wald *z*-distribution approximation.

Analyses were conducted using the R Statistical language (version 4.1.1) [46] on Windows 10 Pro 64 bit (build 19044), using the packages effectsize (version 0.7.0) [47], car (version 3.0.11) [48], Hmisc (version 4.7.0) [49], ggeffects (version 1.1.1) [50], sjPlot (version 2.8.10) [51], report (version 0.5.1.3) [52], rcompanion (version 2.4.15) [53], psych (version 2.1.6) [54], and ggplot2 (version 3.3.5) [55].

## 3. Results

Analysis of socio-demographic data revealed no statistically significant differences in age, gender, and BMI between the study and control groups. In the study group, there were 18 (62.1%) nonsmokers, 5 (17.2%) patients who smoked less than 1 pack per day, and 6 (20.7%) who smoked more than 1 pack per day. In the control group, the distribution of patients in terms smoking was correspondingly 27 (90%), 3 (10%), and 0 (0%).

The application of an independence test showed a significant dependence between the factors group and smoking, with a large effect size, *df* = 2, *V* = 0.38, *p* = 0.010.

A post hoc test revealed a significantly greater proportion of patients who smoked more than 1 pack per day and a lower proportion of nonsmokers in the study group compared with the control group (*p_adj_* = 0.007, *V* = 0.39).

### 3.1. Analysis of Differences in Clinical Data between the Groups

The analyzed clinical characteristics are presented in Table 1. The number of observations *n* reflects the real number of analyses performed.

### 3.2. Analysis of the Relationship between Clinical Parameters, Questionnaire Scores, and Duration of Disease Progression

The results of the analysis of the associations of 18 biochemical parameters with the questionnaire scores and the disease duration factor are shown in Table 2.

### 3.3. Multivariate Analysis Effect of Clinical Parameters on the Depression Factor

A list of seven predictors (HDL, TGA, SOD, 3-NT, GSH, CAT, TRY) was selected for the model based on the significance of differences within these variables between groups. In addition, the same predictors showed significant associations with the questionnaire scores and the duration of depression.

The model fitted with all predictors was characterized by a large variation in inflation factors (vif > 5.0) resulting from high correlations between the parameters TRY vs. SOD (0.87), TRY vs. CAT (0.86), and SOD vs. CAT (0.84). After the elimination of the CAT and TRY parameters, the fitted model had no collinearity (vif < 2.0), so the final model included five clinical parameters: HDL, TGA, SOD, 3-NT, GSH.

A logistic model was fitted to predict depression (binary variable, 1–for study group, 0–for control group.) with HDL, TGA, SOD, 3-NT and GSH (formula: depression~HDL + TGA + SOD + 3-NT + GSH).

The explanatory power of the model was considerable (R^2^_Tjur’s_ = 0.37). The model’s intercept, corresponding to HDL = 0 mg/dl, TGA = 0 mg/dl, SOD = 0, mU/mg protein, 3-NT = 0 nmol/mg protein, and GSH = 0 was -2.53 (95% CI [−7.94, 2.61], *p* = 0.337).

The regression coefficients converted to the scale of OR are presented in Table 3.

**Figure 1:**
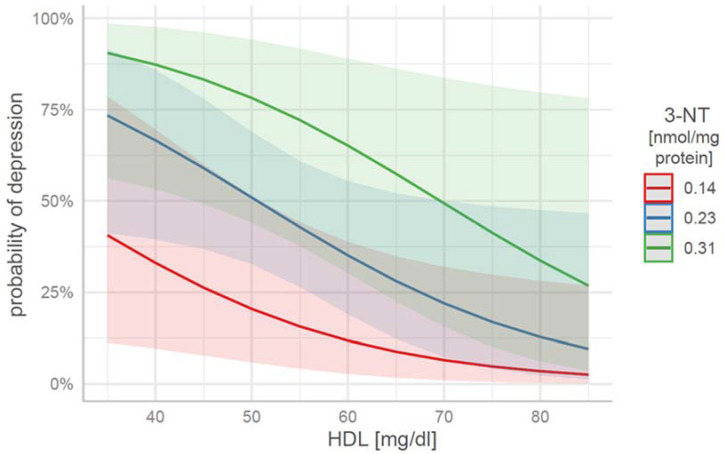
Expected probabilities of depression as a function of the HDL and 3-NT parameters, in the case of 3-NT for values of *M* (0.23) and within one standard deviation, 0.14 (*M*−1*SD*), 0.31 (*M*+1*SD*).

The transformation of the regression coefficients and their plotting on the original probability scale are shown in Figure 1.

The estimated marginal medians (predicted values) for the response variable for significant predictors are presented in Table 4.

## 4. Discussion

The study results support the importance of oxidative stress pathways in the pathogenesis of MDD. Among oxidative stress parameters tested, urine 3-NT seems to have the greatest potential in distinguishing between depressed and healthy people. Urine 3-NT is a product of tyrosine nitration, mediated by RNS—nitrogen dioxide and peroxynitrite anion—and is considered a marker of NO-dependent nitrosative stress. It was previously found to be upregulated in depressed patients [56], in middle-aged women who were more prone to depression [57], adolescents with the depressive disorder [58], and post-stroke patients with depression [59]. All of the abovementioned studies concentrated on 3-NT blood levels. Interestingly, previous studies suggest that nitrotyrosine levels may be reduced by the glutathione precursor, N-acetyl cysteine [60], which is known for its antidepressant properties [61]. Nitrotyrosine has also been associated with neurodegeneration, especially in the dopamine neurons [62], which could be linked to the fact that tyrosine is the biological precursor to dopamine. Urine 3-NT was previously described as a marker of neurodegenerative disorders such as Alzheimer’s, Parkinson’s, and Huntington’s diseases [63]. This could support the concept of the common underpinning of depression and neurodegenerative disorders, as postulated by Maes et al. [10]. Urine 3-NT has also been found to be upregulated in bipolar disorder [64].

Lipid disturbances are frequently observed in patients with mental disorders. This is not surprising, taking into account that lipids constitute as much as 50% of dry brain mass, and the proportion of lipids in brain tissue is the second largest after adipose tissue [65]. Alterations in lipids levels were previously described in different mental disorders, including depression [66]. Lower serum HDL concentrations were found in depressed patients and in depressed men who had attempted suicide [67]. However, in a meta-analysis by Li et al. [68], suicidal attempts in MDD have been linked to lower LDL and total cholesterol levels, but not to alterations in HDL or TG. Lower HDL levels may be characteristic of a subgroup of depressive patients above 40 years old, as described in the meta-analysis of Bharti et al. [69]. Decreased HDL was found in patients with a first episode of MDD [70], MDD and anhedonia [71], and post-stroke depression [72]. A higher LDL/HDL ratio was found in depressive patients [73]. Lower HDL was also associated with an immune-metabolic subtype of depression [74]. In contrast, in the meta-analysis of Shin et al. [75], higher HDL levels were linked to a higher incidence of depression, particularly in women.

The lack of race and geographic diversity in this study it is worth noting, since all study participants were Polish and of Caucasian race. On the other hand, it should be noted that MDD patients received different antidepressant drugs before their inclusion in the study. Hence, it is unclear whether the differences observed between MDD patients and healthy controls are only due to the presence or absence of depressive illness, or if they might be related to the previously received antidepressant treatment.

## 5. Conclusions

The biomarkers research in the field of psychiatry constantly develops, offering a plethora of potential new MDD biomarkers. O&NS and lipid disturbances play an important role in MDD etiopathogenesis [76] and thus constitute a promising source of biomarkers. However, the specificity and sensitivity of any biomarker regarded alone remain almost certainly insufficient. Combined determination of urine O&NS marker 3-NT and blood HDL gives promise as a potential marker of MDD. Nevertheless, the clinical usefulness of such a biomarker combination should be further assessed in a dedicated study. There is a need to construct a validated, multi-dimensional biomarker panel to support the diagnostic process, optimal treatment matching, or outcomes prognosis.

## 6. Limitations

Among the study limitations, the small sample size, the need of controlling for age in the results, and the diversity of antidepressant treatments received by the individuals from the study group should be noted. Moreover, a potentially confounding factor is the higher proportion of smokers in the study group. It is also necessary to admit that some determinants are missing—a few study participants were not screened for all the biological parameters described.

## Figures and Tables

**Table 1:** From the data in Table 1, there were significant differences in all questionnaire scores and in seven biochemical parameters between the study and control groups. The group of depressed patients was characterized by higher mean questionnaire scores, higher mean concentrations of serum TGA and urine SOD, 3-NT, CAT, GSH, and TRY levels, and lower serum HDL concentrations. All significant differences were characterized by large effect sizes.

	Variable	Study Group		Control Group		*p*
		*n*	${\hat{\mu }}_{treatment}$	*n*	${\hat{\mu }}_{control}$	
	Questionnaire scores					
	HAM-D score	29	24.1 (7.1)	30	0.33 (0.7)	<0.001
	Beck score	29	31.9 (11.7)	30	4.3 (4.0)	<0.001
	HAM-A score	29	20.0 (14.0–26.0)	30	0.0 (0–0)	<0.001
Material	Clinical parameters					
serum	Cholesterol, mg/dL	22	175.1 (45.2)	30	180.8 (36.0)	0.630
	HDL, mg/dL	22	50.1 (11.4)	30	60.1 (13.1)	0.005
	TGA, mg/dL	22	105.0 (77.0–137.0)	30	71.0 (56.3–100.8)	0.020
	LDL, mg/dL	22	94.5 (74.3–133.0)	30	98.5 (84.0–116.0)	0.910
	CRP, mg/L	28	1.6 (2.6)	30	1.3 (1.6)	0.550
urine	SOD, mU/mg protein	26	2.2 (1.1)	29	1.5 (0.8)	0.007
	3-NT, nmol/mg protein	26	0.3 (0.2 -0.3)	29	0.2 (0.2 -0.2)	0.001
	AGE, AFU/mg protein	26	60.2 (45.9–74.9)	29	56.6 (41.0–77.4)	0.550
	Amyloid, AFU/mg protein	26	9.1 (7.9–10.5)	29	7.9 (6.6–9.7)	0.080
	AOPP, nmol/mg protein	26	37.8 (32.1–42.2)	29	32.9 (28.3–35.9)	0.054
	CAT, nmol H_2_O_2_/min/mg protein	26	1.0 (0.9–1.3)	29	0.9 (0.8–1.1)	0.010
	GSH, ng/mg protein	26	1.4 (1.3 –1.6)	29	1.2 (1.0–1.5)	0.040
	KN, AFU/mg protein	26	50.6 (34.0–62.2)	29	36.5 (30.9–50.2)	0.090
	NFKN, AFU/mg protein	26	25.9 (18.3–34.6)	29	22.1 (17.2–29.1)	0.260
	GPx, mU/mg protein	26	1.1 (1.0–1.6)	29	1.0 (0.8–1.3)	0.070
	TAC, Trolox umol/mg protein	26	2.2 (1.9–3.0)	29	1.9 (1.6–2.5)	0.130
	TOS, nmol/mg protein	26	20.9 (14.8–25.7)	29	15.4 (10.7–23.4)	0.060
	TRY, AFU/mg protein	26	10.7 (9.2–12.9)	29	7.5 (5.9–9.3)	0.001

**Table 2:** HAM-A, HAM-D, and Beck questionnaire scores and the disease duration factor showed significant associations with serum HDL and TGA and urine SOD, 3-NT, CAT, and TRY concentrations. Urine GSH correlated with the HAM-A score. All significant correlations were characterized by large effect sizes. There was a negative correlation with HDL—an increase in questionnaire scores/duration of disease persistence correlated with a decrease in HDL levels. An additive relationship was found with other parameters—an increase in questionnaire scores/duration of disease persistence correlated with an increase in TGA, SOD, 3-NT, CAT, TRY, and GSH levels. The factor that nearly all questionnaire scores correlated with the same parameters is explained by the fact that the questionnaire scores were highly correlated among themselves (r ≥ 0.80).

Parameter	HAM-D Score			BDI Score			HAM-A Score			Persistence of Depression		
	*n*_pairs_	r_p_ (ρ)	*p*	*n*_pairs_	r_p_ (ρ)	*p*	*n*_pairs_	ρ	*p*	*n*_pairs_	ρ	*p*
Cholesterol, mg/dL	52	−0.03	0.840	52	−0.02	0.891	52	−0.15	0.285	52	−0.02	0.901
HDL, mg/dL	52	−0.33	0.017	52	−0.28	0.042	52	−0.41	0.002	52	−0.35	0.011
TGA, mg/dL	52	0.43	0.002	52	0.37	0.007	52	0.40	0.004	52	0.31	0.023
LDL, mg/dL	52	0.00	0.982	52	0.06	0.682	52	−0.05	0.734	52	0.09	0.544
CRP, mg/L	58	0.18	0.176	58	0.17	0.213	58	−0.09	0.512	58	−0.02	0.859
SOD, mU/mg protein	55	0.33	0.013	55	0.39	0.003	55	0.38	0.004	55	0.40	0.003
3-NT, nmol/mg protein	55	0.37	0.005	55	0.33	0.014	55	0.39	0.004	55	0.40	0.002
AGE, AFU/mg protein	55	0.00	0.963	55	0.02	0.893	55	0.11	0.445	55	0.03	0.802
Amyloid, AFU/mg protein	55	0.22	0.108	55	0.22	0.106	55	0.21	0.117	55	0.26	0.059
AOPP, nmol/mg protein	55	0.22	0.114	55	0.13	0.343	55	0.20	0.140	55	0.21	0.125
CAT, nmol H_2_O_2_/min/mg protein	55	0.27	0.044	55	0.30	0.027	55	0.30	0.028	55	0.30	0.024
GSH, ng/mg protein	55	0.17	0.202	55	0.21	0.119	55	0.27	0.043	55	0.27	0.051
KN, AFU/mg protein	55	0.21	0.189	55	0.17	0.203	55	0.21	0.123	55	0.18	0.193
NFKN, AFU/mg protein	55	0.18	0.193	55	0.14	0.307	55	0.18	0.179	55	0.11	0.437
PX, mU/mg protein	55	0.21	0.133	55	0.26	0.056	55	0.20	0.139	55	0.20	0.134
TAC, Trolox umol/mg protein	55	0.17	0.218	55	0.20	0.134	55	0.20	0.144	55	0.20	0.146
TOS, nmol/mg protein	55	0.19	0.158	55	0.27	0.044	55	0.19	0.174	55	0.20	0.141
TRY, AFU/mg protein	55	0.39	0.003	55	0.40	0.002	55	0.39	0.003	55	0.39	0.002

**Table 3:** From the data in Table 3, it is shown that the parameters TGA, SOD, and GSH had no significant effects on the depression factor. A 1.00 mg/dl increase in the HDL parameter decreased the log odds ratios of depression by 0.07—provided the other predictors were controlled for—as shown here and below. Increasing parameter 3 NT by 1.00 nmol/mg protein increased the log odds ratios of depression by 15.52.

Material	Predictors	Depression			
		log(OR)	*SE*	*z*	*p*
	(Intercept)	−2.53	2.63	−0.960	0.337
serum	HDL	−0.07	0.03	−2.03	0.042
	TGA	0.01	0.01	0.89	0.374
urine	SOD	0.75	0.48	1.57	0.117
	3-NT	15.52	7.55	2.06	0.040
	GSH	0.12	0.33	0.37	0.708

**Table 4:** From the data in Figure 1 and Table 4, patients with HDL < 40 mg/dl and 3-NT > 0.3 nmol/mg protein were more likely to be depressed, whereas patients with HDL > 80 mg/dl and 3-NH < 0.15 nmol/mg protein were less likely to be depressed.

HDL	3-NT	Depression Probability	95% CI ll	95% CI ul
35	0.14	0.41	0.11	0.79
35	0.23	0.73	0.41	0.92
35	0.31	0.91	0.56	0.99
60	0.14	0.11	0.03	0.39
60	0.23	0.35	0.19	0.55
60	0.31	0.65	0.30	0.89
85	0.14	0.03	0.00	0.27
85	0.23	0.10	0.01	0.47
85	0.31	0.27	0.04	0.78

## Data Availability

Not applicable.

## Supplementary Materials

The following supporting information can be downloaded at: https://www.mdpi.com/article/10.3390/jcm12010377/s1.
