# Supplementary material for: Urine 3-Nitrotyrosine and Serum HDL as Potential Biomarkers of Depression
Source: J Clin Med. 2023 Jan 3;12(1):377. doi: 10.3390/jcm12010377 (PMC9821220; doi:10.3390/jcm12010377)
Supplement: Supplementary file 1 [file jcm-12-00377-s001.zip › jcm-2087831-supplementary.pdf]

Supplementary document 1

The document list reagents used for the biochemical assays. All of them were obtained from Sigma-Aldrich:

2,2′-azino-bis(3-ethylbenzothiazoline-6-sulfonic acid) diammonium salt (Cat. No. 10102946001),
5,5′-dithiobis(2-nitrobenzoic acid) (Cat. No. D218200),
acetic acid (Cat. No. 33209-M),
adenochrome (Cat. No. A5752),
chloramine T (Cat. No. 402869),
diethylen- etriaminepentaacetic acid (Cat. No. 17969),
dihydronicotinamide adenine dinucleotide phosphate tetrasodium (Cat. No. NADPH-RO),
epinephrine hydrochloride (Cat. No. E4642),
ethylenediaminetetraacetic acid (Cat. No. ED),
ferrous ion (Cat. No. 450278),
flavin adenine dinucleotide disodium (Cat. No. F8384),
glycerol (Cat. No. G5516),
hydrochloric acid (Cat. No. H1758),
hydrogen peroxide (Cat. No. H1009),
phosphate buffer (Cat. No. P5244),
phosphoric acid (Cat. No. 466123),
potassium iodide (Cat. No. 207969),
potassium persulfate (Cat. No. 216224),
potassium phosphate monobasic (Cat. No. P5379),
potassium thiocyanate (Cat. No. P3011),
pyruvic acid (Cat. No. 107360),
sodium acetate (Cat. No. 241245),
sodium carbonate (Cat. No. S2127),
sodium chloride (Cat. No. S9888),
sodium hydroxide (Cat. No. 06203),
sodium phosphate (Cat. No. 342483),
sodium phosphate dibasic (Cat. No. 94046),
sulfuric acid (Cat. No. 339741),
thioflavin T (Cat. No. T3516),
xylenol orange (Cat. No. 398187).
